# Working Memory Deficit and Attentional Distractibility in Schizophrenia

**DOI:** 10.1192/j.eurpsy.2022.538

**Published:** 2022-09-01

**Authors:** M. Becske, C. Marosi, H. Molnár, Z. Fodor, L. Tombor, G. Csukly

**Affiliations:** Semmelweis University, Department Of Psychiatry And Psychotherapy, Budapest, Hungary

**Keywords:** working memory, schizophrénia, cognitive control, frontal-midline theta

## Abstract

**Introduction:**

Meta-analyses suggest that patients with schizophrenia show deficit in working memory – both verbal and visual – and are more distractible. Working memory disturbances are even regarded as the central deficit in schizophrenia by some researchers. Theta synchronization (especially over fronto-central areas) is related to cognitive control and executive functioning during working memory encoding and retention.

**Objectives:**

The main goal of the study was to gain more understanding of the nature of working memory deficit and attentional distractibility in schizophrenia.

**Methods:**

35 patients with schizophrenia and 39 matched controls were enrolled in our study. Participants performed a modified Sternberg working memory task that contained salient and non-salient distractor items in the retention period. A high-density 128 channel EEG was recorded during the task. Event-related theta (4-7 Hz) synchronization was analyzed during working memory encoding (learning) and retention (distractor filtering) in a later time window (350-550 ms).

**Results:**

Patients with schizophrenia showed weaker working memory performance and increased attentional distractibility compared to the control group: patients had significantly lower hit rates (p < 0.0001) and higher distractor-related commission error rates (p < 0.0001). Theta synchronization was modulated by condition (learning < distractor) in both groups but it was modulated by salience only in controls (salient distractor > non-salient distractor, p[patients] = 0.95, p[controls] < 0.001).

**Conclusions:**

Our results suggest that patients with schizophrenia show diminished cognitive control compared to controls in response to salient distractors. Difficulties in cognitive control allocation may contribute to the behavioral results observed in this study.

**Disclosure:**

No significant relationships.

